# Putative role of immune reactions in the mechanism of tardive dyskinesia

**DOI:** 10.1016/j.bbih.2023.100687

**Published:** 2023-09-23

**Authors:** Anton J.M. Loonen

**Affiliations:** Unit of PharmacoTherapy, -Epidemiology & -Economics, Groningen Research Institute of Pharmacy, University of Groningen, Antonius Deusinglaan 1, 9713AV, Groningen, the Netherlands

**Keywords:** Drug-induced movement disorders, Tardive dyskinesia, AIMS, Habenula, Oxidative stress, Cytokines, Neuroinflammation

## Abstract

The term extrapyramidal disorders is most often used for conditions such as Parkinson's disease or Huntington's disease, but also refers to a group of extrapyramidal side effects of antipsychotics (EPS), such as tardive dyskinesia (TD). After a brief description of some clinical features of TD, this article summarizes the relatively scarce results of research on a possible link between mainly cytokine levels and TD. This data was found by systematically searching Pubmed and Embase. The limitations of these types of studies are a major obstacle to interpretation. After describing relevant aspects of the neuroinflammatory response and the neuroanatomical backgrounds of EPS, a new hypothesis for the origin of TD is presented with emphasis on dysfunctions in the striosomal compartment of the striatum and the dorsal diencephalic connection system (DDCS). It is postulated that (partly immunologically-induced) increase in oxidative stress and the dopamine-dependent immune response in classic TD proceed primarily via the DDCS, which itself is activated from evolutionarily older parts of the forebrain. Neuroinflammatory responses in the choroid plexus of the third ventricle may contribute due to its proximity to the habenula. It is concluded that direct evidence for a possible role of inflammatory processes in the mechanism of TD is still lacking because research on this is still too much of a niche, but there are indications that warrant further investigation.

## Highlights

• Experimental data suggest immune system involvement in tardive dyskinesia.
• Structural and functional changes induced by neuroglia largely mediate this.
• Striosomal striatal compartment neurotoxicity results in tardive dyskinesia.
• Lateral habenula may have a role in causing preferentially orofacial symptoms.
• Inflammatory changes of choroid plexus could affect habenular functioning.

## 1 Introduction

Extrapyramidal disorders include Parkinson's disease and Huntington's disease, but they are also a well-known side effects of drugs used to treat mental illness. The classical antipsychotics in particular are known for it, but the second generation drugs of this pharmacotherapeutic group, contrary to everyone's initial hopes, are certainly not free of these so-called extrapyramidal side effects (EPS) (Correll and Schenk, 2008; Van Harten and Tenback, 2011; O'Brien, 2016; D'Abreu et al., 2018; Caroff, 2022). This article will focus on tardive dyskinesia, which is characterized by abrupt, irregular and uneven muscle contractions, of which the patient is often unaware. It typically takes several months to various years for the symptoms to appear, they are usually less prominent after increasing the dosage and become more severe when abstaining from taking the drug (Kane and Smith, 1982; Caroff, 2022). These aspects must be taken into account when searching for a pharmacological mechanism for the onset of TD. Initially, the theory was that long-term use of drugs that block dopamine D2-type (D2) receptors would increase the sensitivity of these receptors (Margolese et al., 2005; Takeuchi et al., 2022). As a result, when the levels of the antipsychotic become lower, these D2 receptors would be overactivated which would result in the reverse of parkinsonism namely dyskinesia. Although this up-regulation of D2 receptors is indeed observed (Margolese et al., 2005), this mechanism does not explain why it can take months to years for dyskinesia to occur as, in fact, the up-regulation occurs immediately. Another explanation is sought in an impairment of extrapyramidal movement inhibitory neurons, in which oxidative stress could play a role (Lohr et al., 2003; Takeuchi et al., 2022).

This narrative review article is intended to stimulate interest in the possibility that neuroinflammatory processes in striatal striosomes and the lateral habenula (LHb) may contribute to causing TD and that future targeted research is thereby promoted. It describes the evidence from animal and human studies and its limitations that immune factors play a role in this regard. To explain the link between neuroinflammation of the LHb and the development of TD, relevant aspect of the organization of the extrapyramidal system will be summarized from various previous papers and a hypothesis about the mechanism of TD will be presented. It is useful here to distinguish between the classic bucco-linguo-masticatory or orofacial syndrome and peripheral or limb-truncal dyskinesia. For this reason, I will begin with a brief description of the characteristics of TD.

## 2 Tardive dyskinesia

Tardive dyskinesia (TD) is one of the tardive movement disorders attributable to long-term use of dopamine receptor antagonists, such as antipsychotics (Savitt and Jankovic, 2018). These and other movement disorders can occur simultaneously and independently of each other which makes the clinical presentation vary across cases and within a single case over time. As a result, the clinical phenomenology of TD is not unambiguously defined nor can it be determined according to fixed standards in epidemiological studies. Most often, the muscles of the mouth area and face are affected and this is referred to as classical TD (Waln and Jankovic, 2013). Peripheral TD is then the form where the muscles of the neck, trunk and limbs show involuntary movements and classical and peripheral TD probably have a different genetic background (Al Hadithy et al., 2009, 2010). These forms are also called orofacial and limb-truncal TD. For a more detailed description of the clinical manifestations, reference is made here to some of the numerous review articles (Van Harten and Tenback, 2011; Waln and Jankovic, 2013; Savitt and Jankovic, 2018; Hirjak et al., 2019; Caroff, 2022). In respiratory TD, the respiratory muscles and diaphragm are dyskinetic (Yassa and Lal, 1986; Rich and Radwany, 1994; Kruk et al., 1995; Vorre and Lange, 2019). The latter form is usually overlooked in epidemiological studies.

The limited reliability of the results of studies on the epidemiology of and risk factors for TD is not only related to the above diagnostic ambiguity. Also, individual cases usually lack accurate data on antipsychotic loading in the history. Combination therapies are very common and the various treatments of people with psychosis are usually not well recorded. At the same time, it is already known from early studies that a relationship (otherwise nonlinear) exists with cumulative exposure to dopamine antagonists: the duration of use, the potency of the drug and the dosage at which it was applied (Kane and Smith, 1982). Precisely as seen with other side effects, this does seem to involve a flattening of the curve: the incidence decreases with advancing years of exposure (Kane and Smith, 1982; Tarsy and Baldessarini, 2006). The difference between various antipsychotics is most evident in relatively short-term studies of the incidence of TD when using first-versus second-generation antipsychotics (Correll and Schenk, 2008; O'Brien, 2016; Carbon et al., 2017, 2018). It does deserve mention that a large-scale randomized pragmatic study found no significant differences between the classic perphenazine and the second-generation agents studied (Caroff et al., 2011). Some uncertainty exists about having greater susceptibility to motor side effects as a risk factor for getting and having TD (Kane and Smith, 1982; Miller et al., 2005; Solmi et al., 2018), in part because it may also be related to hereditary pharmacokinetic or other effect-determining factors (Loonen et al., 2019; Caroff, 2022). A consistently found risk factor is advanced age (Miller et al., 2005; Carbon et al., 2017; O'Brien, 2016; Caroff, 2022). Many other risk factors have been cited (Solmi et al., 2018; Caroff, 2022), but the research findings are often less unambiguous than for age.

## 3 Biomarkers for neuroinflammation and tardive dyskinesia

A single reviewer (AJML) systematically examined Pubmed and Embase. The design and results are described in Appendix A according to the PRISMA statement (Liberati et al., 2009). Details of the species and immune factors studied as well as the main conclusions of the identified studies are given in Table 1, Table 2.

**Table 1:** Identified animal studies about relationship between substances linked to immune response and tardive dyskinesia.

Source	Species	Immune factor (IF) studied	Drug studied	Results	Reason excluded
Bishnoi et al. (2008a)	Male rats	TNF-α (striatum)	HAL, clozapine or risperidone	HAL > CLZ HAL > RIS	included
Bishnoi et al. (2008b)	Male rats	TNF-α (striatum)	HAL	dose-dependently	included
Bishnoi et al. (2009)	Male rats	TNF-α (striatum)	HAL	dose-dependently	not complete original
Bishnoi et al., 2011^a^	Male rats	TNF-α (striatum)	HAL ± curcumin	drug ≠ control	included
Blanchet et al. (2018)	Monkeys, capuchin	none (only kinases, putamen)	HAL or clozapine (CLZ)	HAL ≠ CLZ	no IF measured
Datta et al. (2016)	Male rats	IL-1β, TNF-α, IL-6 (striatum)	HAL ± lycopene	drug ≠ control	included
Grover et al. (2013)	Male rats	IL-1β, TNF-α (striatum)	HAL ± pioglitazone HAL ± fenofibrate	drug ≠ control IL-1β: drug ≠ control	included
Jamwal & Kumar (2018)	Rats	IL-1β, TNF-α, IL-6 (striatum)	HAL ± lycopene	drug ≠ control	not complete original
Lewitus et al. (2011)	Mice (±KO)	TNF-α	HAL	wildtype ≠ knockout	included
Mezzomo et al. (2022)	Male rats	IL-1β, TNF-α, IL-6 (striatum)	HAL ± Isoflavones	drug ≠ control	included
Peroza et al. (2016)	Male rats	IL-1β, TNF-α, IL-6, IL-10, IFN-γ (striatum)	HAL, risperidone (RIS)	IL-1β, IFN-γ: HAL > RIS IL-6, TNF-α, IL-10: HAL = RIS	included
Rahi et al. (2022)	Rats of either sex	none	HAL ± Filgrastim (GCSF-α agonist)	drug suppressed TD	No other IF measured
Sonego et al. (2018)	Male mice	IL-1β, TNF-α, IL-6, IL-10 (striatum)	HAL ± cannabidiol	IL-1β, TNF-α, IL-10: drug ≠ control	included
Sonego et al. (2021)	Male mice	IL-1β, TNF-α, IL-6, IL-10	HAL ± cannabidiol	IL-1β, TNF-α: drug ≠ control	included
Soung et al. (2018)	Male rats	TNF-α, IL6 (striatum)	RES ± L-Theanine	drug ≠ control	included
Thakur et al. (2015)	Male rats	IL-1β, TNF-α (cortex, striatum)	HAL ± candesartan HAL ± lisinopril	both drugs ≠ control	included

Abbreviations: CLZ – clozapine; GCSF - granulocyte colony stimulating factor; IF – immune factor; IL – interleukin; HAL – haloperidol; RES – reserpine; RIS – risperidone; TNF – tumor necrosis factor.

a Not identified with the developed search strategy (tardive dyskinesia or TD not used).

**Table 2:** Identified studies in humans about relationship between substances linked to immune response and tardive dyskinesia.

Source	Number of patients	Immune factors studied	Results	Reason to exclude
An et al. (2015)	TD = 48, NTD = 45	IL-2, IL-6, IL-8	IL-2, IL-6: TD ≠ NTD	included
Boiko et al. (2017)	TD = 71, NTD = 109	IL-1β, TNF-α, IL-3, IL-6, INF-γ	IL-6: TD ≠ NTD	this conference abstract was included
Bošković et al. (2013)	TD = 6, NTD = 46	*TNF* (−308G > A genotype)	TD = NTD	included
Choi et al. (2020)	TD = 105, NTD = 175	*IL-10* (−1082G/A genotype)	TD ≠ NTD	included
Kang et al. (2009)	TD = 83, NTD = 126	*TNF-α* (−308G/A genotype) *IL-10* (−1082G/A genotype)	TD = NTD TD = NTD	this conference abstract was included
Lanning et al. (2017)	TD = 72, NTD = 106	Neurexin-1 (NRXN1 genotypes)	TD = NTD	no IF studied
Li et al., 2021	TD = 61, NTD = 61	TLR4 signal pathway	TD ≠ NTD	entirely different design (ex vivo model)
Liu et al. (2012)	TD = 45, NTD = 45	IL-2	TD ≠ NTD	included
Rajan et al. (2018)	TD = 18, NTD = 152	IL-6, hsCRP, hepcidin	TD = NTD	only patients with bipolar disorder
Rapaport and Lohr (1994)	TD = 13, NTD = 12	IL-2 (SIL-2R)	TD ≠ NTD	included
Sun et al. (2013)	TD = 372, NTD = 412	*IL-10* (−592C > A genotype)	TD = NTD	included
Tian et al. (2014)	TD = 46, NTD = 43	TNF-α	TD = NTD (p = 0.05)	included
Tiwari et al. (2009)	Schizophrenia = 233	None (DARPP-32 genotypes)	TD = NTD	no IF studied
Uludag et al., 2023	TD = 77	ApoA1, ApoB, IL-2, IL-6, IL-8, TNF-α	TNF-α predicts best	entirely different design
Wang et al. (2012)	TD = 350, NTD = 410	*TNF-α* (−308A/G genotype)	TD = NTD	included
Wu et al. (2022)	TD = 121, NTD = 118	IL-2, IL-6, IL-8, TNF-α	TNF-α: TD ≠ NTD	included

DARPP-32 - Dopamine- and cAMP-regulated phosphoprotein, Mr 32 kDa; hsCRP - high sensitivity C-Reactive Protein; IF – Immune factor; NTD - patients not with TD; TD - patients with TD; SIL-2R - soluble interleukin-2 receptor.

### 3.1 Findings in experimental studies

#### 3.1.1 Interleukin 1 beta (IL-1β)

Interleukin 1 beta (IL-1β) is a cytokine protein that in humans is encoded by the *IL1B* gene (loc: 2q14.1) and that belongs to the interleukin 1 family. It is produced by activated macrophages as a proprotein, which is proteolytically converted to its active form by caspase 1. This pro-inflammatory cytokine is an important mediator of the inflammatory response, and is further involved in a variety of cellular activities, including cell proliferation, differentiation and apoptosis.

Increased (in comparison to control animals who were only exposed to the vehiculum used) striatal levels of IL-1β were invariably found in rodent models of haloperidol-induced orofacial dyskinesia (Grover et al., 2013; Peroza et al., 2016; Thakur et al., 2015; Datta et al., 2016; Sonego et al., 2018, 2021; Mezzomo et al., 2022). Both dyskinesia and IL-1β levels were reduced when the animals received concurrent antioxidant treatment. IL-1β levels correlated with the frequency of orofacial movements (Peroza et al., 2016; Sonego et al., 2018, 2021). However, striatal mRNA levels were not altered (Sonego et al., 2018).

Only a conference abstract describing peripheral IL-1β levels in humans was found (Boiko et al., 2017). No significant difference was found in serum levels of IL-1β in patients with schizophrenia and with TD (n = 71) compared to patients without TD (n = 109).

#### 3.1.2 Interleukin 6 (IL-6)

Interleukin 6 (IL-6) is both a pro-inflammatory cytokine and an anti-inflammatory myokine (a peptide or protein secreted or released from skeletal muscle cells). In humans, it is encoded by the *IL6* gene (loc: 7p15.3). It is secreted by macrophages and then triggers the acute phase response. In addition, osteoblasts secrete IL-6 to stimulate osteoclast formation. Smooth muscle cells in the wall of blood vessels also produce IL-6 as a pro-inflammatory cytokine. The role of IL-6 as an anti-inflammatory myokine is mediated by its inhibitory effects on TNF-α and IL-1 and its activation of IL-1ra and IL-10.

Although rodent studies are somewhat inconsistent, all but two in mice (Sonego et al., 2018, 2021) reported increased striatal IL-6 levels in haloperidol (Peroza et al., 2016; Datta et al., 2016; Mezzomo et al., 2022)-induced or reserpine (Soung et al., 2018)-induced orofacial dyskinesia. However, in two studies (Peroza et al., 2016; Sonego et al., 2021) co-treatment with antioxidants (isoflavones, cannabidiol) did not reduce IL-6 levels and in two studies (Peroza et al., 2016; Sonego et al., 2018) striatal IL-6 levels did not correlate with the frequency of orofacial dyskinesia. Sonego et al. (2018) also measured striatal mRNA expression in mice and saw that it was increased by haloperidol and significantly less during cannabidiol co-treatment. There were significant correlations between striatal mRNA levels and severity of a measure of orofacial dyskinesia.

Results from human studies are contradictory. Both marginally significant increases (Boiko et al., 2017; with tardive dyskinesia (TD) = 71/without tardive dyskinesia (NTD) = 109; conference abstract), significant decreases (An et al., 2015; TD = 48/NTD = 45) and the existence of no difference (Wu et al., 2022; TD = 121/NTD = 118) have been reported.

#### 3.1.3 Interleukin 10 (IL-10)

Interleukin 10 (IL-10) is an anti-inflammatory cytokine that is a member of the class-2 cytokines. In humans, interleukin 10 is encoded by the *IL10* gene (loc: 1q32.1) and is produced primarily by monocytes and, to a lesser extent, lymphocytes. IL-10 inhibits the induction of secretion of the pro-inflammatory cytokines TNF-α, IL-1β, IL-12, and IFN-γ by myeloid immune cells.

Sonego et al. (2018, 2021) reported that in mice, neither haloperidol nor cannabidiol affected striatal IL-10 levels, but the combination increased the levels in one of these studies (Sonego et al., 2018). Peroza et al. (2016) found that in rats both haloperidol and risperidone reduced striatal IL-10 levels, while in this experiment risperidone did not cause orofacial dyskinesia, and in the haloperidol group the levels did not correlate with the severity of dyskinesia.

The *IL-10* -1082G/A polymorphism (rs 1800896) was genotyped in schizophrenia patients by Kang et al. (2009; TD = 83/NTD = 126; conference abstract) and Choi et al. (2020; TD = 105/NTD = 175). The only significant difference was observed by the latter authors who found that the A allele frequency was higher in the TD group. Sun et al. (2013; TD = 372/NTD = 412) found that the allele and genotype frequencies of rs1800872 (−592A/C) were not significantly different.

#### 3.1.4 Tumor necrosis factor alpha (TNF-α)

Tumor necrosis factor alpha (TNF-α) is the commonly used but actually former name for the adipokine and cytokine: tumor necrosis factor (TNF, cachexin or cachectin). The *TNF* gene (loc: 6p21.33) encodes for a transmembrane protein of 233 amino acid residues (mTNF), from which a soluble cytokine (sTNF) is released via proteolytic cleavage after substrate presentation. mTNF is present on a variety of cell types, but mainly on monocytes/macrophages. Activation of TNF has direct and indirect pro-inflammatory effects, but also causes direct cellular effects.

Of all the cytokines, striatal TNF-α levels is still the most frequently studied in animal experiments. After multiple administrations to rats of haloperidol (Bishnoi et al., 2008a, 2008b, 2011; Grover et al., 2013; Thakur et al., 2015; Peroza et al., 2016; Datta et al., 2016; Sonego et al., 2021; Mezzomo et al., 2022), risperidone (Peroza et al., 2016) and reserpine (Soung et al., 2018) striatal TNF-α levels invariably increased, with only in the case of risperidone this exposure not being associated with orofacial dyskinesia (Peroza et al., 2016). In the study by Bishnoi et al. (2008a), clozapine and risperidone did produce orofacial dyskinesia to some extent, but did not increase striatal TNF-α levels. Concomitant exposure to substances with antioxidant properties reduced or prevented the increase in both (Bishnoi et al., 2011; Grover et al., 2013; Thakur et al., 2015; Soung et al., 2018 Sonego et al., 2021; Mezzomo et al., 2022). In the study by Peroza et al. (2016), striatal TNF-α levels did not correlate with the frequency of dyskinetic movements. The latter was also observed in mice, but in this case there was a correlation between expression of *TNF* mRNA and this frequency (Sonego et al., 2018). Activation of microglia was also observed in the latter study. In their second study Sonego et al. (2021) did find a significant relationship between striatal TNF-α levels and the frequency of the dyskinetic movements. Moreover, *TNF-α* knockout mice exhibited an exacerbation of haloperidol-induced orofacial dyskinesia, which also persisted longer than in wild-type mice (Lewitus et al., 2011).

In three studies, individuals with schizophrenia were genotyped for rs1800629 (−308G/A) which is located on the promoter region of the *TNF* gene and regulates expression of TNF-α (Kang et al., 2009 (TD = 83/NTD = 126; conference abstract); Wang et al., 2012 (TD = 350/NTD = 410); Bošković et al., 2013 (TD = 6/NTD = 46)). The results were always negative. Tian et al. (2014; TD = 46/NTD = 43) and Wu et al. (2022; TD = 121/NTD = 118) found (marginally) lower TNF-α blood levels in individuals with schizophrenia with and without TD, but (Boiko et al., 2017; TD = 71/NTD = 109; conference abstract) found no significant differences.

#### 3.1.5 Interleukin 2 (IL-2)

Interleukin 2 (IL-2) is a pro-inflammatory cytokine for which is encoded by the *IL2* gene (loc: 4q27). Activated lymphocytes express IL-2 and IL-2 receptors, promoting proliferation of their own clone. In addition to T lymphocytes, natural killer cells (NK cells) also secrete IL-2 and have IL-2 receptors.

No studies with laboratory animals were found about this interleukin. The findings in humans with schizophrenia are contradictory. Both significantly higher serum IL-2 levels (An et al., 2015; TD = 48/NTD = 45) and significantly lower levels (Liu et al., 2012; TD = 45/NTD = 45) are reported in people with TD, while Wu et al. (2022); TD = 121/NTD = 118) found no significant differences. In the study Liu et al. (2012), serum levels did not correlate with measured severity of TD. An old study by Rapaport and Lohr (1994) found higher serum soluble interleukin 2 receptor (SIL-2R) levels in 13 patients with TD compared with 12 patients without TD.

#### 3.1.6 Interleukin 8 (IL-8)

Interleukin 8 (IL-8) or chemokine (C-X-C motif) ligand 8 (CXCL8) is a pro-inflammatory chemokine produced by macrophages/microglia and other cell types. The *CXCL8* gene (loc: 4q13.3) encodes for a precursor peptide of 99 amino acids, which is then cleaved into different isoforms. It induces chemotaxis in target cells and stimulates phagocytosis once they arrive.

No animal experimental studies were discovered about this cytokine either. In humans with schizophrenia, the results of both An et al. (2015; TD = 48/NTD = 45) and Wu et al. (2022; TD = 121/NTD = 118) are negative in terms of detecting differences in measured serum levels of IL-8.

#### 3.1.7 Interferon gamma (IFN-γ)

Interferon gamma (IFN-γ) belongs to the class II interferons, which are produced by T lymphocytes based on the *IFNG* gene (loc: 12q15). It activates macrophages/microglia and thus acts pro-inflammatory.

Striatal IFN-γ levels were elevated in chronic treatment with both haloperidol and risperidone, but only haloperidol revealed orofacial dyskinesia in this experiment (Peroza et al., 2016). The frequency of dyskinetic movements correlated significantly with striatal IFN-γ levels in the first case.

Boiko and colleagues (2017; TD = 71; NTD = 109; conference abstract) found no significant differences between the serum levels of people with schizophrenia with or without TD.

### 3.2 General criticism of the value of these studies

Besides the fact that the number of studies is still small, there are also important limitations in terms of their reliability due to the methods used, which must be considered when interpreting the results.

#### 3.2.1 Animal experimental studies

The most studied species is the rat. In a few studies mice are studied and almost always only male individuals. Dyskinesia is usually induced by several weeks of daily administration of haloperidol with or without combination with the drug under study. The authors often only claim antioxidant effects of these drugs, but they (*e.g.,* cannabidiol; Martinez Naya et al., 2023) often have other important pharmacological effects as well. In these studies, the agents tested were administered together with haloperidol or reserpine, so only the influence on the development of TD was examined. Almost always they concentrate on orofacial dyskinesia. The cytokine level is measured in tissue homogenates at the end of the period of haloperidol administration. Only one study also measured cytokine expression (by measuring mRNA). For an animal pharmacological study, weeks of treatment with haloperidol is quite lengthy, but compared to human tardive dyskinesia with an incidence of roughly 3–5% per treatment year (Caroff, 2022), this duration is minimal. This may mean that the phenomena in the animal model differ in fundamental ways from those in the development of tardive dyskinesia in humans.

#### 3.2.2 Human studies

There are genetic association studies and studies of peripheral cytokine levels in individuals with schizophrenia with and without TD. The sample size of the first type of studies is (quite) small. Usually the presence of dyskinesia is measured with the abnormal involuntary movement scale (AIMS), without specifying the version used. The unmodified AIMS has substantial limitations that make its validity and reliability low (Loonen and van Praag, 2007; Loonen et al., 2000), but this is not a major concern when only the prevalence and not the severity of TD is considered. The study never distinguishes between orofacial and peripheral dyskinesia and respiratory dyskinesia is not measured at all. Dyskinesia also occurs spontaneously in people with schizophrenia (Fenton, 2000; Lee et al., 2008; Pappa and Dazzan, 2009; Koning et al., 2010; Walther and Mittal, 2017), their siblings (McCreadie et al., 2003; Koning et al., 2010) and – although the true incidence is debated (Ticehurst, 1990) – in older individuals (Blanchet et al., 2004; Merrill et al., 2013). By definition, the included patients are treated with dopamine 2 antagonists (antipsychotics) which by themselves might have immune modulating effects via these receptors (Xia et al., 2019; Loonen, 2023). Moreover, antipsychotics have other side effects involving inflammatory processes, such as hepatotoxicity (Todorović Vukotić et al., 2021) and metabolic syndrome (Leonard et al., 2012; Fang et al., 2020). The historical “neuroleptic load” (which is: dose equivalent _*_ duration of use _*_ weight factor for type of drug) cannot be determined and is expected to be unequal for those with and without TD and between the studies. Schizophrenia is also associated with altered cytokine levels in treatment-naïve individuals (Dunleavy et al., 2022), and in the considered studies often greater differences exist between patients and healthy volunteers than between patients with and without TD (not shown). The relative contribution of primary peripheral and primary cerebral inflammatory processes is unclear.

### 3.3 Preliminary conclusions

Due to the sometimes contradictory findings and the limitations mentioned in the above subsection, no firm conclusions can be derived from the research already conducted. IL-1β may play a role in mediating orofacial TD in rodent models, but it is not expressed to an increased extent in the animal's striatum. This leaves room for speculation that it is generated elsewhere. With IL-6, the relationship with current orofacial dyskinesia is considerably less certain, but an association does exist in the mouse with the expression of this cytokine in the striatum. However, species differences may be involved. The results of human research are too contradictory for any conclusions. That the anti-inflammatory IL-10 has an inhibitory effect on the mechanism causing orofacial TD may be an attractive thought, but the evidence from animal and human genetic research is in fact razor-thin. For TNF-α, the findings in animal experiments are roughly similar to those for IL-1β. With some exceptions there is an association between the current presence of orofacial dyskinesia and TNF-α levels in the rat striatum, but now the severity of dyskinesia does not correlate with the striatal level of this cytokine. In the mouse, however, a relationship does exist between severity of dyskinesia and expression of TNF and activation of microglia is also seen. In humans, the results of genetic studies and serum level measurement are almost entirely negative. For IL-2, IL-8, only human studies of the association between serum levels and TD exist, the results of which are also negative. In fact, too little research has been done on IFN-γ to draw even tentative conclusions.

Hence, striatal IL-1β levels correlate with the presence of orofacial dyskinesia but are probably not primary in this regard. The relationship between striatal TNF-α levels and dyskinesia is weaker, but the evidence for a pathogenetic role is stronger. The evidence in the case of striatal IL-6 levels is still too contradictory to interpret and the evidence about INF-γ levels is too limited, but positive for a relationship with actual dyskinesia. The limited and contradictory findings with IL-2, IL-8 and IL-10 make it too uncertain for drawing conclusions.

## 4 Neuroinflammatory processes of the central nervous system

The above description of cytokines does not distinguish between their role in the central nervous system (CNS) and the rest of the body. This does not make much sense either, since the days when the CNS was thought to be hermetically sealed off from the “actual” immune system are many years behind us. Better to emphasize the interactive relationships between the neuronal, endocrine and immunological regulatory systems (Bottaccioli et al., 2022). Here, although there are barriers between the CNS and the rest of the body, there is also intensive communication across these boundaries (Miller and Raison, 2016). In this communication, cytokines play an important role (Soria et al., 2018). They are produced in the CNS mainly by microglia, but also by astroglia, neurons and peripheral immune cells (lymphocytes and macrophages), the latter after crossing the blood brain barrier (BBB) (Bernaus et al., 2020). Cytokines bring about their pleiotropic effects by binding to specific receptors that are also expressed by all the aforementioned cell types. They cause local inflammatory responses along this pathway but also have a great influence on the structure and function of neuronal networks (Wolf et al., 2017; Sancho et al., 2021). In a recent article, I suggested that neuroinflammation probably also affects neurocommunicative processes within the DDCS (Loonen, 2023). Another border between the periphery and the brain that may be bridged by immunocommunication is the blood cerebrospinal fluid barrier (BCSFB) and especially that in the choroid plexus where most of the cerebrospinal fluid (CSF) is produced (Demeestere et al., 2015; Tumani et al., 2017; Cui et al., 2021; Gião et al., 2022; Santos-Lima et al., 2022). The barrier consists of a single layer of epithelial cells attached to each other by so-called tight junctions (Demeestere et al., 2015). The stromal compartment contains fenestrated capillaries, fibroblasts and different types of immune cells. These include stromal macrophages, which belong to a different population from the epiplexus macrophages on the CSF side of the BCSFB that in turn are distinct from the microglia in the brain parenchyma (Cui et al., 2021). Whether cytokines can pass the healthy BCSFB is not entirely clear to me. While the epiplexus macrophages play their own role in the immunological surveillance of the CSF (Cui et al., 2021), it is not excluded that they may still respond to peripheral immunological messages and then produce cytokines themselves. This includes penetrating peripheral immune cells (T cells and neutrophil granulocytes) (Demeestere et al., 2015; Santos-Lima et al., 2022). The latter obviously plays a role in inflammatory processes with damage to the BCSFB (Gião et al., 2022; Santos-Lima et al., 2022). In this context, it deserves mentioning that enlargement of the choroid plexus has also been observed in neuropsychiatric disorders such as schizophrenia (Zhou et al., 2020) and was especially true in patients with TD (Li et al., 2021).

Among the most important cytokines for neuroinflammation in the brain are TNF-α, IFN-γ, IL-1β and IL-6 (Tang and Le, 2016; Linnerbauer et al., 2020; Jia et al., 2021). Within the CNS these pro-inflammatory cytokines are mainly produced by microglia (Tang and Le, 2016; Jia et al., 2021). Microglia are undoubtedly the most important native immune cells of the CNS (Wolf et al., 2017; Mattei and Notter, 2020; Deng et al., 2020). They perform a role as macrophages and further have a task in pruning away neuronal connections in developing and adult brain (Kettenmann et al., 2013; Lenz and Nelson, 2018), function in a process called ‘surveillance’ (Nimmerjahn et al., 2005; Eyo and Wu, 2019; Deng et al., 2020), but can also be activated to a pro-inflammatory M1 or an anti-inflammatory M2 state (Boche et al., 2013; Tang and Le, 2016; Bernaus et al., 2020; Rahimian et al., 2021; Sancho et al., 2021). IFN-γ activates microglia to the M1 state in which they produce the aforementioned pro-inflammatory cytokines as well as superoxide, reactive oxygen species (ROS) and nitric oxide (NO) (Tang and Le, 2016; Jia et al., 2021). Astrocytes can also be induced to produce TNF-α (Chung and Benveniste, 1990; Bernaus et al., 2020). In the M2 activation state, microglia are brought by the anti-inflammatory IL-4/IL-13 (alternate activation) and by IL-10/transforming growth factor β (TGF-β) (acquired de-activation) with the aim of attenuating the pro-inflammatory response again, and in this state, on balance, they have a neuroprotective effect (Tang and Le, 2016; Jia et al., 2021; Chai et al., 2022).

In addition to activation by, for example, pathogen entry into the CNS (Boche et al., 2013; Bernaus et al., 2020; Mattei and Notter, 2020; Deng et al., 2020), the immune response can also be initiated from the periphery (Miller and Raison, 2016; Kim and Won, 2017; Gonçalves and De Felice, 2021; Bottaccioli et al., 2022). This means, on the one hand, that forms of psychological stress through activation of an immune response, for example, via the autonomic nervous system, could trigger a neuroinflammatory process in the CNS and, on the other hand, that tissue damage elsewhere in the body through the initiation of an immune response could also be responsible for a neuroinflammatory process in the CNS (Loonen, 2023).

## 5 Neuroanatomical background of extrapyramidal side effects

**Fig. 1:**
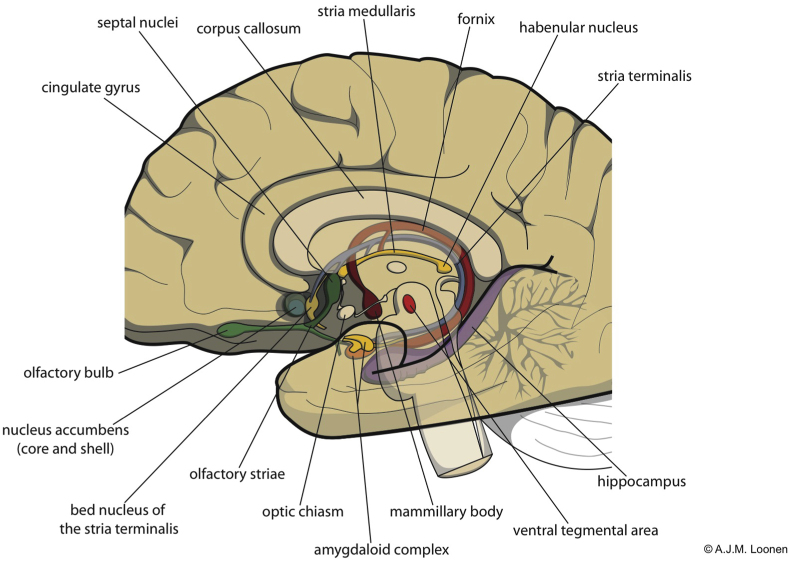
Open-worked and schematic representation of the human brain showing some possibly lesser-known anatomical structures that play a role in regulating motor components of behavior.

The neuroanatomical structures and relationships mentioned in this section have been discussed in detail in several previous publications which are cited beneath. Please refer to these writings for further details and substantiation with literature data. Because some of the readers may have difficulty with some parts of the description, in the following some of the important anchor points are first mentioned in a general introduction (Fig. 1).

### 5.1 Gross anatomy of important brain structures

The human brain consists of a brain stem, cerebellum and forebrain (Loonen, 2021). The forebrain consists of two hemispheres connected by mainly the anatomical thalamus. In the anatomical thalamus, from ventral to dorsal can be distinguished: subthalamus, hypothalamus, dorsal thalamus and epithalamus. The dorsal thalamus is by far the largest and is referred to simply as “thalamus” by most clinicians. The epithalamus is adjacent to the third cerebral ventricle and consists of stria medullaris, habenula and epiphysis (the latter not shown in Fig. 1). Both hemispheres consist of a lining of cerebral cortex and an inner subcortex. The dorsal striatum and globus pallidus are the largest subcortical basal ganglia, but are not shown in Fig. 1. The former is subdivided into caudate nucleus and putamen. The second into pars interna (GPi) and pars externa (GPe). The cerebral cortex is divided into 5 lobes of which the lobe adjacent to the frontal bone occupies half of the total. The temporal lobe contains the oldest parts of the forebrain: the hippocampus and the amygdaloid complex. This last structure consists of a corticoid part and a ganglionic part, the latter also being called “extended amygdala.” It consists of an interconnected nuclear amygdala adjacent to the corticoid part and a further inward bed nucleus of the stria terminalis. The nucleus accumbens is the ventral and medial part of the striatum. It is not readily visible macroscopically in humans. The corresponding ventral pallidum is not shown in Fig. 1. The activity of the forebrain is regulated by monoaminergic (dopamine, 5-hydroxytryptamine, norepinephrine) and cholinergic pathway systems, which flow from the upper part of the brainstem to the basal forebrain, striatum and cerebral cortex and, in the case of acetylcholine, to the dorsal thalamus. Of these, the figure shows only the ventral tegmental area from which dopaminergic fibers flow to the striatum and frontal cortex. Feedback from the forebrain proceeds to the midbrain via a ventral pathway in the opposite direction, but also via a dorsal pathway via the habenula in the epithalamus: the dorsal diencephalic connection system (DDCS).

### 5.2 Evolutionary history of the extrapyramidal system

**Fig. 2:**
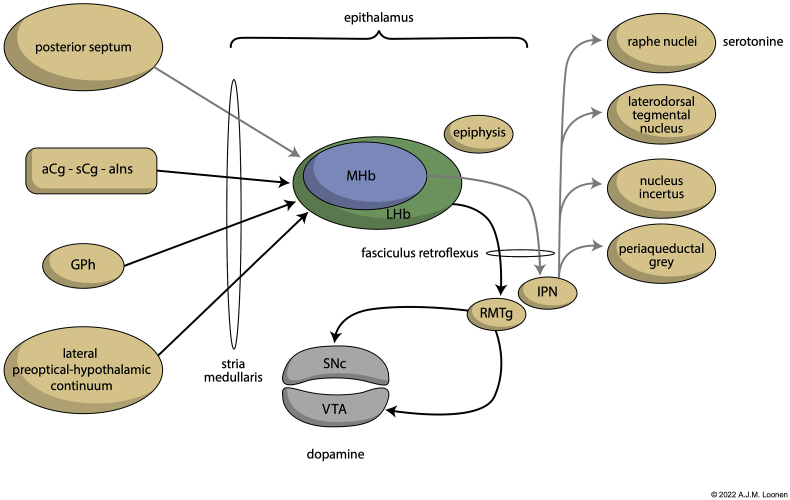
Anatomy of the central part of the dorsal diencephalic connection system (DDCS). The aCg-sCg-aIns is a continuum of limbic cortical areas running from aCg through sCg to aIns which also receives input from ventral extrapyramidal re-entry circuits. For more details consult Loonen and Ivanova (2022). aCg – anterior cingulate cortex; aIns – anterior insular cortex; epiphysis – pineal gland; GPh – habenula-projecting pallidum; IPN – interpeduncular nucleus; LHb – lateral habenula; MHb – medial habenula; RMTg – rostromedial tegmental nucleus; sCg – subgenual cingulate cortex; SNc – Substantia nigra, pars compacta; VTA – ventral tegmental area.

Usually authors of articles on movement disorders limit themselves to considering the dorsal extrapyramidal system, but this is not entirely correct. The other two divisions, the amygdaloid and the ventral extrapyramidal system, are at least as important for the development of movement disorders such as tardive dyskinesia. They originated much earlier during vertebrate evolution: about 560 million years ago (mya) and 370 mya, and are part of what I have called the primary and secondary forebrain, respectively (Loonen and Ivanova, 2018a, 2018b, 2022). The primary forebrain initiates adequate emotional responses and the secondary forebrain regulates their intensity and the readiness to exhibit them. Important for this article is their link with the dorsal diencephalic connection system or DDCS (Loonen and Ivanova, 2019, 2022). Through this DDCS, which includes the habenula in the epithalamus, the forebrain provides input to the midbrain (Fig. 2). The connections between the habenula and midbrain have hardly changed since the first vertebrates which evolved 560 mya. They regulate the activity of cholinergic and monoaminergic centers in the midbrain (Loonen and Ivanova, 2022). By modulating the activity of the ascending dopaminergic fibers to the striatum, the DDCS also plays a role within the dorsal extrapyramidal system. For further description, please refer to the cited review articles.

### 5.3 The dorsal division of the extrapyramidal system

An educationally focused review article (Loonen and Ivanova, 2013) provides a highly simplified outline of the human dorsal extrapyramidal system. For a more detailed description of the anatomy and physiology of this system, reference is made to recent articles on the mechanisms of dyskinesia (Loonen et al., 2019) and dystonia (Loonen and Ivanova, 2021). Referring to these older papers, it is sufficient to summarize these at this point.

#### 5.3.1 Cortico-striato-thalamo-cortical circuits

**Fig. 3:**
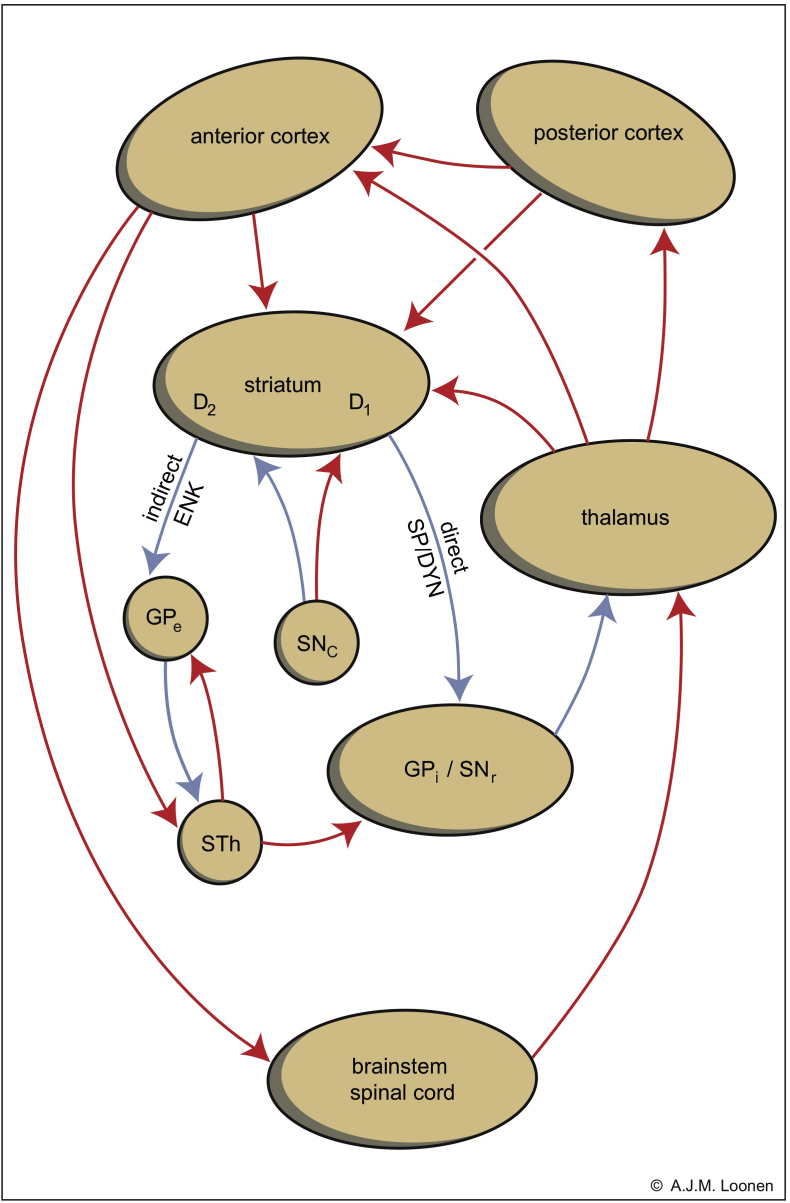
Indirect (left) and direct (right) striatopallidal pathways representing […] of the CSTC circuits. Corticostriatal neurons synapse with two types of inhibitory medium-sized spiny projection neurons (MSNs): D2 receptor-carrying, enkephalin-containing, indirect pathway MSNs and D1 receptor-carrying, substance P and dynorphin-containing, direct pathway MSNs. Stimulation of both D1 and D2 receptors results in disinhibition of thalamocortical neurons and therefore increased cortical output. D1 = dopamine D1 receptor-expressing medium spiny neurons; D2 = dopamine D2 receptor-expressing medium spiny neurons; GPe = globus pallidum externa; GPi = globus pallidus interna; SNc = substantia nigra pars compacta; STh = nucleus subthalamicus; SNr = substantia nigra pars reticulata. Red = stimulatory, blue = inhibitory. (For interpretation of the references to colour in this figure legend, the reader is referred to the Web version of this article.)

The main structure for dyskinesia is a set of more or less parallel and converging circuits that run from the cerebral cortex through the putamen (as the motor part of the striatum) to the globus pallidus, pars interna or the substantia nigra, pars reticulata (GPi/SNr) and then through the thalamus to the frontal cerebral cortex: the cortico-striato-[…]-thalamo-cortical circuits or CSTC circuits (Fig. 3) (Loonen et al., 2019; Loonen and Ivanova, 2021). The sign -[…]- herein represents connections by GABAergic Medium-sized Spiny projection Neurons (MSNs) from the striatum to the GPi/SNr structure along a direct or indirect pathway. The MSNs of the indirect pathway carry dopamine D2-type (D2) receptors and are enkephalin-containing. MSNs of the direct pathway carry D1-type (D1) receptors and contain dynorphin and substance P in addition to GABA. Indirect pathway MSNs are particularly sensitive to neurotoxic changes. Activation of the direct pathway results in activation of the cortical terminus of the CSTC circuit and activation of the indirect pathway in inhibition thereof.

#### 5.3.2 Striatal matrix and striosomal compartments

The striatum is not uniform in construction. It consists of a continuous matrix containing scattered patches or striosomes with a different structure (Loonen et al., 2019; Loonen and Ivanova, 2021). The CSTC circuits all pass through the striatal matrix and originate in all parts of the cerebral cortex (including the frontal lobe). The striosomes, however, receive input only from the limbic medial prefrontal cerebral cortex (Eblen and Graybiel, 1995) and corticoid amygdala (Ragsdale and Graybiel, 1988). They also contain the cell bodies of both types of MSNs, but these regulate the activity of ascending dopaminergic pathways from the midbrain. D1 receptor-carrying MSNs run directly to the dopaminergic nuclei in the midbrain (Crittenden et al., 2016). The other MSNs project to specific areas in the pallidum (GPh in Fig. 2) and from there to the lateral habenula (LHb) (Stephenson-Jones et al., 2016; Hong et al., 2019). The LHb regulates the activity of both dopaminergic and serotonergic (5-hydroxytryptamine, 5-HT) pathway systems (Loonen and Ivanova, 2019). Also within striosomes, enkephalin-expressing indirect pathway (D2 receptor-carrying) MSNs occur more at the edges and D1 receptor-bearing MSNs, which are in the majority in striosomes, hence more in the center (Prensa et al., 2009; Brimblecombe and Cragg, 2017).

#### 5.3.3 Ascending dopaminergic neurons

Dopaminergic fibers have their cell bodies in the midbrain from a dorsal tier formed by a continuum corresponding to the A9-A8-A10 regions (containing the substantia nigra, pars compacte (SNc) and the ventral tegmental area (VTA)) and the ventral layer below with bulges in the substantia nigra, pars reticulata (SNr) (Prensa et al., 2009). Dopaminergic fibers of this ventral tier have a different biochemical composition and it is precisely these fibers that are lost in Parkinson's disease (Prensa et al., 2009). They also have a different distribution in the striatum. Dopaminergic fibers from the dorsal tier run only to the matrix, whereas fibers from the ventral tier serve both the matrix and striosomal compartments (Kreitzer, 2009; Prensa and Parent, 2001; Prensa et al., 2000, 2009). The release of ventral tier dopaminergic varicosities occurs more in the center of the striosomes (Prensa et al., 2009). Dopaminergic fibers from the ventral tier could well be very important in the development of tardive dyskinesia (Loonen et al., 2019).

### 5.4 Summary

The main element of the dorsal extrapyramidal system are the convergent cortico-striatal-thalamo-cortical circuits that flow from all parts of the cerebral cortex to the frontal cerebral cortex. These circuits use only the matrix compartments of the striatum. Within each of these circuits exists a direct and indirect pathway, the first part of which is formed by MSNs with mutually distinguishable characteristics. Through the striosomal compartment of the striatum flow pathways that come from limbic prefrontal and corticoid amygdala areas and regulate the activity of ascending dopaminergic pathways. From these striosomes, inhibitory D1 receptor-bearing MSNs project directly to dopaminergic nuclei in the midbrain. D2 receptor-bearing MSNs project to pallidal areas and from there signals via the lateral habenula increase the activity of ascending dopaminergic pathway systems. The dopaminergic nuclear systems in the midbrain have two layers. From the dorsal tier, dopaminergic fibers run to the CSTC circuits and cholinergic interneurons of the matrix. From the ventral tier, they run to both the matrix and striosomes. By activation of striosomal D1 receptor-bearing MSNs from the direct pathway, they provide feedback inhibition of the activity of ascending dopaminergic pathways.

## 6 Some new ideas for a possible mechanism for tardive dyskinesia

### 6.1 Neurotoxicity due to oxidative stress

It is known that in a resting state mainly inhibitory striatal D2 receptors are activated which therefore keeps the output of the extrapyramidal system low. When dopamine release in the striatum is increased for whatever reason, D1-receptors are also activated, thereby increasing the output of the extrapyramidal system (Kreitzer, 2009). This also takes place in the striosomal compartment so that the increased output directly or indirectly via the LHb results in inhibition of the activity of the ascending dopaminergic midbrain neurons. When antipsychotics chronically antagonize D2 receptors, also in striosomes the D2 receptor-carrying indirect MSNs become overactive. Via the LHb pathway, this will result in compensatory increases in the activity of ascending dopaminergic pathways, which target both striatal matrix and striosomes. The increased release leads to increased uptake of dopamine in MSNs and to increased oxidative stress and neurotoxic damage, to which striosomal D2 receptor-bearing MSNs in particular appear to be susceptible (Rikani et al., 2014; Morigaki and Goto, 2017; Bergonzoni et al., 2021). This cellular damage can also result in activation of microglia and thereby initiate an immune response.

### 6.2 Immunomodulation by dopamine

Actually probably more important in the latter context is the role of dopamine as a modulator of the immune system (Pinoli et al., 2017; Xia et al., 2019; Thomas Broome et al., 2020). Dopamine provides some of the communication between neurons and neuroglia and other immune cells, as well as between immune cells themselves (Thomas Broome et al., 2020). The precise role of dopamine in this regard is not yet fully crystallized. Different types of dopamine receptors are expressed on astrocytes and microglia (Xia et al., 2019; Thomas Broome et al., 2020). Thereby, the five different subtypes of the D1-type (DRD1, DRD5) and D2-type (DRD2, DRD3, DRD4) dopamine receptors have a mutually different function that differs from the activating and inhibitory effect, respectively, in the extrapyramidal system (Xia et al., 2019; Thomas Broome et al., 2020). These receptors are expressed on astrocytes, (especially activated) microglia and T helper cells (Th cells) (Xia et al., 2019; Thomas Broome et al., 2020), but the effects cannot be summarized unambiguously. Moreover, dopamine plays a similar role in several other peripheral immune cells (Pinoli et al., 2017). All in all, it is quite conceivable that changes in dopamine metabolism both in schizophrenia and its treatment with antipsychotics have consequences for the immune response in the CNS.

### 6.3 Dorsal diencephalic connection system

In addition to being an indirect pathway from the striosomal compartment, the involvement of the dorsal diencephalic connection system (DDCS) with the habenula as a connection hub can be hypothesized in yet other ways. MSNs from striosomes project to glutamatergic neurons in the pallidum which in turn project fibers to the LHb (Stephenson-Jones et al., 2016; Hong et al., 2019). Both the striosomal compartment of the striatum and, partly indirectly, the DDCS receive much input from the medial prefrontal cerebral cortex and the corticoid amygdala (Ragsdale and Graybiel, 1988; Loonen and Ivanova, 2018a, 2022). The DDCS regulates the activity of ascending monoaminergic pathways. When output from the extrapyramidal system is chronically too low, compensatory mechanisms are also activated within the DDCS regulatory system. Here, insufficient activation of the primary and secondary parts of the forebrain due to extrapyramidal hypoactivity also leads to additional stimulation via the DDCS. Because this phylogenetically much older regulatory system is more involved in orofacial and axial muscle movements than in movements of younger muscle groups of the extremities, one would expect more orofacial TD than peripheral TD as a result. It can be speculated upon that peripheral dyskinesia arises primarily from the imbalance between inhibitory and activating extrapyramidal pathways beginning in the putamen, whereas the classical form of dyskinesia may find its basis more in a dysregulation in the dorsal diencephalic connective system within the habenula.

### 6.4 Choroid plexus

When considering a possible special role of the DDCS in the development of classical (orofacial) TD, the proximity of the choroid plexus of the third ventricle to the epithalamus should also be noted. An important consideration here is that structural (possibly neuroinflammatory) changes in schizophrenia also involve the choroid plexus (Lizano et al., 2019; Zhou et al., 2020; Li et al., 2021). As described above, neuroinflammatory changes in the choroid plexus may affect the appearance of cytokines and antibodies in the CSF in its vicinity. They can then also penetrate into the surrounding brain parenchyma, because the ependymal border with the CSF in the ventricles is not so tight. This field is still relatively unexplored, but it is certainly quite conceivable that it plays a role in preferential influence of the DDCS in all kinds of neuropsychiatric disorders in general, but certainly also in TD.

### 6.5 Summary

Based on the foregoing, it can be postulated that classical and peripheral dyskinesia are the two different forms how chronic blocking of dopamine D2 receptors in the striosomal compartment of the putamen plays out. It can be argued that peripheral dyskinesia arises primarily from the imbalance between inhibitory and activating extrapyramidal pathways beginning in the matrix of the putamen, whereas the classical form of dyskinesia may have its basis more in a dysregulation in the DDCS within the habenula. In both cases, the neurochemical basis may be found in an increase in oxidative stress which may also be due to neuroinflammatory processes.

## 7 Discussion and conclusions

This narrative review article sought to present arguments for a possible importance of inflammatory processes in the mechanism of TD and to draw attention to the possibility that these inflammatory responses might be localized in the epithalamus. Research on this is still too much of a niche for direct evidence to exist. This can be concluded from the lack of sufficient data on the possible relationship of cytokine levels or gene polymorphisms with the prevalence of TD. Data do exist that indirectly indicate an inflammatory component. Most important among these is that it has long been known that schizophrenia (and, for that matter, also mood disorders) involve neuroinflammation (Sperner-Unterweger and Fuchs, 2015; Müller et al., 2015; Mednova et al., 2022) and that dopamine itself plays a role in modulating the immune response (Pinoli et al., 2017; Xia et al., 2019; Thomas Broome et al., 2020). So antipsychotics are used in disorders where there is neuroinflammation anyway, and they can enhance it by increasing the release of dopamine. Where these neuroinflammatory processes take place has not been adequately investigated. Studies in animal models of TD have found increased levels of IL-1β, IL-6 and TNF-α in tissue homogenates of the striatum. Increased expression of IL-6 and TNF-α also correlates with the number of mouth movements, which may indicate that on-site production is responsible for the increase and symptoms. Within the boundaries of the striatum, the striosomal compartment might well play a greater role than the matrix compartment, which could help explain the preference for orofacial musculature in classic tardive dyskinesia. Objectionable to the interpretation of the animal data remains the aggressiveness of the intervention leading to the development of orofacial dyskinesia in animal models. The neuroinflammatory changes leading to the development of TD need not take place in the striatum. Immune activation outside the central nervous system has important im-plications for neuroinflammatory processes *in cerebro* (Miller and Raison, 2016; Erickson and Banks, 2018; Gonçalves and De Felice, 2021; Loonen, 2023). It is possible that the elevated IL-1β is not produced in the striatum, because the corresponding mRNA levels are not elevated there (Sonego et al., 2018). Compact and phylogenetically old structures such as the amygdaloid and habenuloid complexes and the hippocampus are very suitable targets for peripherally produced cytokines and chemokines. Inflammatory responses of the choroid complex could be the instigator of exposure to cytokines and therewith activation of neuroglia in nearby areas of brain tissue. I would like to call special attention to the possible influence on the function of the epithalamus located in the immediate vicinity of choroid plexus of the third ventricle. The role that the DDCS plays in regulating the activity of cholinergic and ascending monoaminergic pathway centers in the midbrain makes this at all a good candidate to generate behavioral components of neuroinflammation.

## Funding source

This research did not receive any specific grant from funding agencies in the public, commercial, or not-for-profit sectors.

## Declaration of competing interest

None.

## Data Availability

Data will be made available on request.
