# Supplementary material for: Putative role of immune reactions in the mechanism of tardive dyskinesia
Source: Brain Behav Immun Health. 2023 Sep 23;33:100687. doi: 10.1016/j.bbih.2023.100687 (PMC10550815; doi:10.1016/j.bbih.2023.100687)
Supplement: Multimedia component 1 [file mmc1.pdf]

Appendix A

Putative role of Immune Reactions in the Mechanism of Tardive Dyskinesia: systematic review of relevant studies

Anton J.M. Loonen

Abstract:
Background: An unstructured search of various databases found 19 research reports on appropriate measures of activation of immune processes in tardive dyskinesia. It is questionable whether this adequately explored the field.
Objectives: What is the relation for human patients with schizophrenia (P) or in experimental (animal) models of tardive dyskinesia (P), of immunological measures (I) and the occurrence (O) or severity (O) of tardive dyskinesia?
Data Sources: Searched were the databases of Pubmed (NIH/NLM platform) and Embase (Elsevier platform) without applying additional filters.
Study Selection: Only a single reviewer (AJML) selected appropriate studies with and without imposing further restrictions in the search.
Data Extraction Methods: A single reviewer extracted the relevant data from each publication and described them in the main text of the article.
Data Synthesis: The number of publications found was considered too small and their content too heterogeneous to arrive at a formal aggregation of findings.
Limitations: The limitations of the studies found are described in a separate paragraph from the main text of the article.
Conclusions: Found were 12 animal studies and 11 patient studies that met the question. Because of their great heterogeneity and methodological shortcomings, they do not provide a sufficient basis for firm conclusions.

Introduction

Rationale: Tardive dyskinesia (TD) is an extrapyramidal movement disorder occurring with and probably due to antipsychotics and other antagonists of dopamine receptors of the D2 subtype. They are accompanied by fairly rapid involuntary movements of the face, tongue and jaw (orofacial or classical TD), of the trunk and extremities (limb-truncal or peripheral TD) or of the muscles involved in breathing (respiratory TD). Although this has been relatively little studied, there is evidence that the immune response plays some role in the development of this movement disorder. To track down publications, an initial search of Pubmed (NIH/NLM platform) was conducted primarily using the keywords: cytokines (or cytokine) and the MESH term "tardive dyskinesia." The keyword cytokines was also replaced by chemokines (or chemokine) because some immune factors are also chemokines and tardive dyskinesia results in intense muscle activity. The titles of the articles found were manually screened. Further variation was also made on the term cytokine with the words interleukin and the umbrella MESH term "Intercellular Signaling Peptides and Proteins," as well as the MESH term "cell adhesion molecules" and the MESH term "acute phase proteins." Cell Adhesion Molecules play a role in leukocyte penetration through the blood-brain barrier and C-Reactive Protein is an indicator of inflammatory responses somewhere in the body. The titles found were manually reviewed for suitability. The MESH term "Tardive Dyskinesia" was not varied so as not to confuse it with other drug-induced dyskinesia or chorea. Then, using the original search terms cytokine and tardive and dyskinesia, the Embase, PsycINFO and Web of Science (WoS) databases were examined. The Embase database includes abstracts of presentations at scientific meetings and PsycINFO and WoS publications outside the medical field. Examining Pubmed found 17 articles and Embase found 2 additional conference abstracts. PsycINFO and WoS yielded no new publications. These 19 abstracts were searched, studied and summarized in a paper. They form the base set for the first version of the article. Based on

the above experiences, it was decided to use only Pubmed and Embase for the systematic review.
Objectives: A PICO was compiled from the discovered publications: What is the relation for human patients with schizophrenia (P) or in experimental (animal) models of tardive dyskinesia (P), of suitable immunological measures (I) and the occurrence (O) or severity (O) of tardive dyskinesia? Suitable immunological measures (I) should include (blood, CSF, brain tissue) levels of cytokines, chemokines, cell adhesion molecules, or acute-phase reactants.

Methods
Protocol and registration: Given the many uncertainties, the criteria on which to select publications were not predefined and neither established in a protocol. In retrospect, the most important criterion was that the abbreviation TD had to refer to tardive dyskinesia. Also, the measured substance had to play a role within the context of the study in the communication of the immune response, but intracellular communication mechanisms were excluded as they are not specific enough.
Eligibility criteria: No specific filters were used in selecting the records. In the search engines consulted, at least the title was given in English, but the article itself could also be in Dutch, French or German.
Information sources: Pubmed (NIH/NLM platform) and Embase (Elsevier platform) were searched, for the last time on April 18th, 2023.
Search: Medline (NIH/NLM platform) was searched with:
("Tardive Dyskinesia"[Mesh] OR "tardive dyskinesia*"[tiab] OR TD[tiab])
AND
("Cytokines"[Mesh] OR cytokine*[tiab] OR chemokine*[tiab] OR interleukin*[tiab] OR interferon*[tiab] OR "tumor necrosis factor*"[tiab] OR TNF[tiab] OR "Adipokines"[Mesh] OR adipokine*[tiab] OR adipocytokine*[tiab] OR "Fibroblast Growth Factors"[Mesh] OR "fibroblast growth factor*"[tiab] OR "fibroblast growth regulatory factor*"[tiab] OR "TGF-beta Superfamily Proteins"[Mesh] OR "TGF-beta superfamily protein*"[tiab] OR "transforming growth factor beta"[tiab] OR "Cell Adhesion Molecules"[Mesh] OR "cell adhesion molecule*"[tiab] OR "cellular adhesion molecule*"[tiab] OR "leukocyte adhesion molecule*"[tiab] OR CAM[tiab] OR "Acute-Phase Proteins"[Mesh] OR "acute-phase protein*"[tiab] OR "acute phase reactant*"[tiab] OR "C-Reactive Protein"[Mesh] OR "C-reactive protein"[tiab] OR CRP[tiab])
Embase (1974 – Present) was searched with:
('tardive dyskinesia'/exp OR 'tardive dyskinesia*':ti,ab OR TD:ti,ab)
AND
('cytokine'/exp OR cytokine*:ti,ab OR chemokine*:ti,ab OR interleukin*:ti,ab OR interferon*:ti,ab OR 'tumor necrosis factor*':ti,ab OR TNF:ti,ab OR adipokine*:ti,ab OR adipocytokine*:ti,ab OR 'fibroblast growth factor*':ti,ab OR 'fibroblast growth regulatory factor*':ti,ab OR 'transforming growth factor beta':ti,ab OR 'TGF-beta superfamily protein*':ti,ab OR 'cell adhesion molecule'/exp OR 'cell adhesion molecule*':ti,ab OR 'cellular adhesion molecule*':ti,ab OR 'leukocyte adhesion molecule*':ti,ab OR CAM*:ti,ab OR 'acute phase protein'/exp OR 'acute phase protein*':ti,ab OR 'acute phase reactant*':ti,ab OR 'C reactive protein'/exp OR 'C reactive protein':ti,ab OR CRP:ti,ab)
By a single researcher (AJML), manually by considering the title and abstract, those records were selected for further consideration. This specifically excluded initially identified articles in which the search term TD did not refer to tardive dyskinesia. The manual search was then repeated again on the results of a search in which the term td had been replaced by "dyskinesia (TD)"[tiab] in Pubmed and "dyskinesia (TD)":ti,ab in Embase. A pdf of the articles that belonged to the identified records were all searched for, downloaded and reviewed piece by piece. One article found in the unstructured initial search was not present in the results because the term tardive dyskinesia did not appear in it (more commonly neurotoxicity). This article was also added to the final selection. The results are shown in the flow diagram (Figure A1) and in Tables 1 and 2.

Data collection process: Because the publications found after classification as animal studies or investigations in humans were limited in number and heterogeneous in design, the results were summarized descriptively by a single investigator (AJML) in the text of the main paper. Quantitative data extraction was not yet considered useful.
Data items: In addition to the substances of the immune system that provide communication between cells themselves, studies were also included on the receptors and genes encoding these substances. In addition to the terms listed under search, in human studies the selection criterion considered was having the diagnosis of schizophrenia. Consideration was given to specifical adding nuclear factor kappa-light-chain-enhancer of activated B cells (NF-κB) to the search terms and describing the results separately. This was abandoned because it refers to an intracellular mechanism not only involved in the immune response. The search term dyskinesia was not included because it mainly covers studies in patients with Huntington's or Parkinson's disease. Other terms found that were not included in the search terms are: dopamine- and cAMP-regulated neuronal phosphoprotein (MW 32 kDa, DARPP-32) also known as Protein phosphatase 1 regulatory subunit 1B (PPP1R1B), hepcidin, mitogen-activated protein kinase (MAPK), peroxisome proliferator activated receptor gamma (PPARγ), and toll-like receptor (TLR).
Risk of bias in individual studies: The limitations of the found studies according to the personal judgment of the researcher are summarized and discussed in a separate section of the main document.
Summary measures: significance of differences ($p < 0.05$).
Planned methods of analysis: none.
Risk of bias across studies: The main form of bias may have arisen in the manual screening of the titles and abstracts of the initially identified records to arrive at a set of possible candidates by only a sole investigator. Also, the decision not to expand the search strategy to include the above search terms may have resulted in bias. Two conference abstracts were found not to be included in full papers, which may be related to the negative results. This may have resulted more often in publication bias.
Additional analyses: none.

Results

Study selection: A total of 23 studies, including 12 that described experiments in animals and 11 in humans, were identified for inclusion in the review. Searching in Pubmed yielded 866 records and in Embase 1885 titles. After removing records in which TD did not refer to tardive dyskinesia and removing papers that clearly did not meet the criteria, manual screening of titles and abstracts and combining the records yielded 31 papers. The full text of these 31 citations was searched and further content examined. Five of them were found to be the abstract of a conference paper, with the more detailed final paper also present in the set in 2 cases. In 4 studies, the studied entity could not ultimately be named as an immune factor. In one study of the effects of a Granulocyte Colony Stimulating Factor agonist, this is formally the case, but no further indicators of the immune response itself were measured. Two of the identified studies in humans were so different in design from the rest that it was decided not to include these results. One human study did not study people with schizophrenia; a different immune process may be at play in people with bipolar disorder than in people with schizophrenia. One article originally found in the free search did not appear in either Pubmed or Embase selection. It turned out that the title and abstract only talked about neurotoxicity and not mentioned tardive dyskinesia (TD). This article is also added to the selection. For a flow diagram and final selection, see Figure A1 and Tables 1 and 2.
Characteristics of included studies: As can be seen in Tables 1 and 2, the different studies differ quite a bit in design and/or parameters studied. Therefore, it was not considered useful (yet) to summarize and analyze these results.
Risk of bias within studies: The limitations and sources of bias of the final selected studies are described in the main text of this article. Specific deficiencies associated with the

methods used in individual articles were deemed to be beyond the scope of this article at this time.
Results of individual studies: The results of the final selected studies are described in the main text of this article.
Synthesis of results: Because the selected studies were quite different in terms of study design and parameters studied, it was decided to refrain from summarizing and mutually analyzing individual study results.
Risk of bias across studies: To reduce the risk of bias in identifying records, an attempt was made to always include lowly potentially relevant terms that are as high or higher up in the tree (MESH or EMTREE). Another form of bias arises in selecting records when manually selecting potentially interesting articles based on title and abstract. In each case, another specific search was made of articles where at least the term - dyskinesia (TD) - was mentioned in title or abstract.
Additional analysis: not applicable.

Discussion
Summary of evidence: The evidence is summarized in the main text of this article.
Limitations: The main limitation is that this study was conducted entirely by only one reviewer, the heterogeneity of the studies is relatively high, and no study has yet examined the relationship between the immune response at the level of the dorsal diencephalic connection system (habenula) and tardive dyskinesia.
Conclusions: Data are widely available on IL-1β, IL-6, IL-10 and TNF-α and to some extent on IL-2, IL-8 and IFN-γ.

Funding
This research received no specific grant from any funding agency in the public, commercial, or not-for-profit sectors.
Acknowledgements
Advice for constructing the search strategies was obtained from the UMCG Central Medical Library information specialist Peter G. Braun.
