# Supplementary material for: Putative role of immune reactions in the mechanism of tardive dyskinesia
Source: Brain Behav Immun Health. 2023 Sep 23;33:100687. doi: 10.1016/j.bbih.2023.100687 (PMC10550815; doi:10.1016/j.bbih.2023.100687)

Figure A1

PRISMA 2020 flow diagram for identifying publications about relationship between substances linked to immune response and tardive dyskinesia

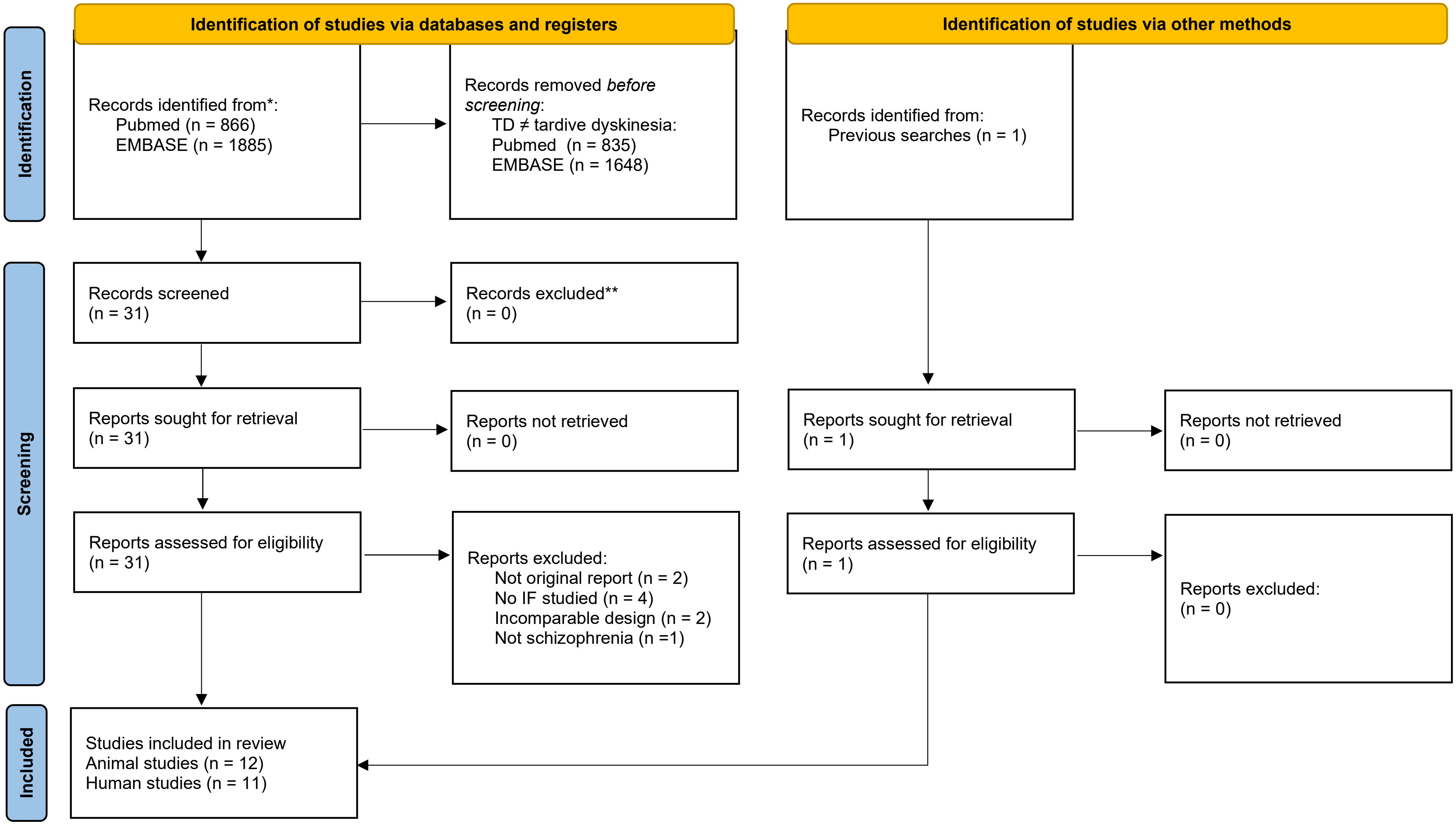

Supplement: Multimedia component 2 [file mmc2.pdf]
